# Next-Generation Strategies to Encounter Antimicrobial Resistance (AMR): From Lariocidin to Gene Editing and Nanotechnology-Based Approaches

**DOI:** 10.3390/molecules31132395

**Published:** 2026-07-07

**Authors:** Ilknur Yilmaz, Bekir Mustafa Yoğurtçu, Samson Aisida, Enes Baki Ezer

**Affiliations:** 1 Department of Molecular Biology and Genetics, Faculty of Engineering and Natural Sciences, İstanbul Atlas University, Istanbul 34408, Turkey; 2 Department of Molecular Biology and Genetics, Graduate School of Science & Engineering, Yildiz Technical University, Istanbul 34220, Turkey; bmustafayogurtcu@gmail.com; 3 Department of Physics and Astronomy, University of Nigeria, Nsukka 410001, Nigeria; samson.aisida@unn.edu.ng; 4 Department of Bioengineering, Yildiz Technical University, Istanbul 34220, Turkey; 5 Graduate School of Science and Technology, Niigata University, Nishi-ku, Niigata 950-2181, Japan; enesezer0571@gmail.com

**Keywords:** AMR, novel antibiotics, lariocidin, CRISPR-Cas systems, nano delivery system

## Abstract

The escalation of antimicrobial resistance (AMR) represents a serious global threat to public health, with AMR-associated mortality estimated to increase by 70% by 2050. As pathogens evolve through enzymatic inactivation, target modification, efflux-mediated clearance, biofilm formation, and broader genetic adaptation, conventional therapies are increasingly compromised, while the antibiotic development pipeline remains critically constrained by high discovery and development costs, weak commercial incentives, and the escalating complexity of resistance mechanisms. This review comprehensively synthesizes advanced pharmacological and biotechnological innovations designed to circumvent these entrenched resistance mechanisms. We highlight the development of novel therapeutic classes, particularly lariocidin, which disrupts bacterial protein synthesis via a previously unexploited ribosomal-binding site. Moreover, we critically evaluate molecular interventions, emphasizing CRISPR/Cas-based gene silencing and genome editing as precise tools to neutralize specific resistance determinants, such as the *mecA* gene in methicillin-resistant *Staphylococcus aureus* (MRSA). Concurrently, we explore the integration of engineered nanoparticles to revitalize existing antimicrobials by overcoming biofilm barriers, improving drug solubility, and enabling targeted delivery. Collectively, mastering the evolving AMR landscape requires a multidimensional framework that seamlessly integrates these novel molecular targets with advanced rapid diagnostics and robust international governance.

## 1. Introduction

**Figure 1:**
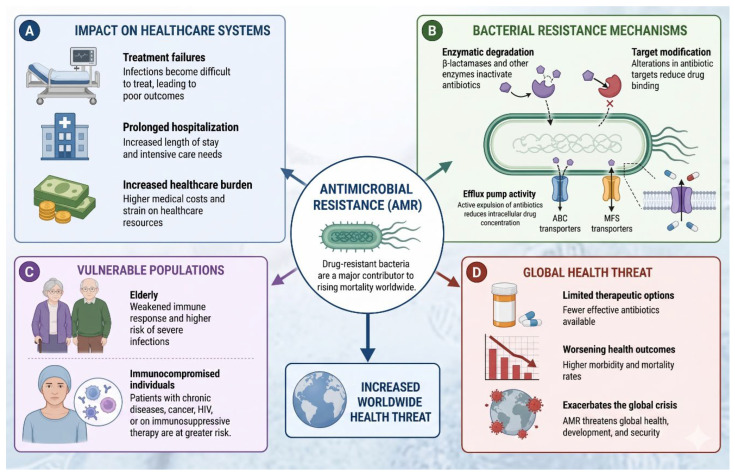
Global impact of antimicrobial resistance. (**A**) The figure outlines key consequences for healthcare systems. (**B**) Depicts major bacterial resistance mechanisms. (**C**) Vulnerable populations, particularly the elderly and immuno-compromised individuals, are disproportionately affected. (**D**) AMR limits therapeutic options and exacerbates the global health crisis.

Bacterial infections remain formidable threats to global public health, imposing substantial burdens of morbidity and mortality, particularly in vulnerable populations. The escalating challenge of AMR has propelled the search for novel therapeutic strategies to the forefront of medical research. International health organizations, including the World Health Organization (WHO), consistently emphasize the critical need to address the dwindling efficacy of existing antimicrobials against resistant microorganisms [1,2]. Recent analyses have directly linked 1.27 million deaths to AMR and estimate that it caused 5 million deaths globally in 2019 alone [3]. Projections based on trends from 1990 to 2021 suggest a potential increase in AMR-associated mortality, threatening healthcare systems and disproportionately affecting aging populations. Despite advances in therapy, infections such as bloodstream infections and ventilator-associated pneumonia continue to challenge treatment efficacy, especially in severe cases and resource-limited settings (Figure 1). Addressing these challenges demands the accelerated discovery and development of next-generation antibiotics with novel mechanisms of action, alongside comprehensive antimicrobial stewardship and global surveillance efforts. Rather than isolating specific pathogens or singular interventions, this review provides an integrated analysis of the current AMR landscape by focusing on novel chemical scaffolds, such as lariocidin, with advanced biotechnological countermeasures like CRISPR-Cas silencing and nanocarrier delivery. Through this synthesis, we outline a comprehensive therapeutic and diagnostic framework necessary to outpace bacterial adaptation.

## 2. Bacterial Resistance Mechanisms and Counteracting Strategies

Bacterial resistance to antibiotics is a multifaceted phenomenon involving diverse molecular strategies that enable pathogens to evade antimicrobial activity. As the global burden of multidrug-resistant (MDR) infections continues to rise, pharmaceutical research has increasingly focused on developing targeted therapeutic approaches capable of overcoming these resistance mechanisms. Bacteria employ a variety of survival strategies, including enzymatic antibiotic degradation, active efflux, reduced membrane permeability, and modification of antimicrobial targets. Addressing these highly adaptive defense systems requires innovative and mechanism-specific interventions [4].

Enzymatic inactivation remains one of the most prevalent and effective bacterial resistance mechanisms. β-Lactamases hydrolyze the β-lactam ring, thereby rendering β-lactam antibiotics such as penicillins and cephalosporins ineffective [5]. In addition to enzymatic degradation, bacteria frequently limit intracellular drug accumulation through active efflux systems and reduced membrane permeability. Resistance-Nodulation-Division (RND) efflux pumps actively expel antimicrobial agents, lowering intracellular concentrations below therapeutic levels. To address this challenge, researchers have designed structurally modified antibiotics that evade efflux pump recognition, while efflux pump inhibitors (EPIs) are being investigated as adjunctive agents to enhance antibiotic retention within bacterial cells [6].

Outer membrane impermeability and porin loss represent additional barriers, particularly in Gram-negative pathogens. Innovative “Trojan horse” strategies have emerged as effective solutions to bypass these obstacles. Cefiderocol, a siderophore-cephalosporin conjugate, exemplifies this approach by exploiting bacterial iron-uptake systems to facilitate active transport across the outer membrane [7]. By utilizing nutrient acquisition pathways, cefiderocol achieves high intracellular concentrations even in strains with impaired porin function. Bacterial survival may also result from alterations in the molecular targets of antibiotics, reducing drug-binding affinity while preserving essential cellular functions [8]. To circumvent such resistance mechanisms, novel antimicrobial agents have been engineered to engage alternative and highly conserved target sites. Gepotidacin, for example, avoids classical fluoroquinolone resistance through a distinct dual-targeting interaction with bacterial DNA gyrase and topoisomerase IV. Likewise, lariocidin binds to a previously unexploited ribosomal site, maintaining activity against strains resistant to conventional protein synthesis inhibitors. By targeting novel and conserved cellular structures, these next-generation therapeutics reduce the likelihood of resistance development and expand the available arsenal against MDR pathogens [9].

In recent years, the global burden associated with ESKAPE pathogens has continued to rise, reinforcing their central role in the AMR crisis. The term “ESKAPE pathogens” refers to a group of clinically significant bacteria that are responsible for most hospital-acquired infections and are particularly adept at evading the effects of antimicrobial agents. This group includes *Enterococcus faecium*, *S. aureus*, *Klebsiella pneumoniae*, *Acinetobacter baumannii*, *Pseudomonas aeruginosa*, and *Enterobacter* species [10]. These organisms are strongly associated with MDR, increased virulence, and persistence in healthcare environments, especially in intensive care units where vulnerable patient populations are concentrated. ESKAPE pathogens remain leading contributors to healthcare-associated infections worldwide and are frequently implicated in severe conditions such as bloodstream infections, ventilator-associated pneumonia, and complicated wound infections [11]. Their clinical significance is further underscored by their association with elevated mortality rates, largely driven by treatment failures and prolonged hospital stays. Current evidence indicates that resistance among ESKAPE pathogens is not only persistent but also escalating. Many strains now exhibit extensive drug resistance (XDR) or even pan-drug resistance (PDR), significantly narrowing available therapeutic options. To standardize the characterization of resistance profiles discussed throughout this review, we refer to the internationally recognized criteria: MDR is defined as non-susceptibility to at least one agent in three or more antimicrobial categories; XDR indicates non-susceptibility to at least one agent in all but two or fewer antimicrobial categories, and PDR refers to non-susceptibility to all agents in all available antimicrobial categories. Alarmingly, resistance has been reported even against recently developed antimicrobial agents, highlighting the rapid adaptive capacity of these organisms [12]. This trend underscores a critical gap between the pace of antimicrobial development and the evolution of bacterial resistance mechanisms. Recognizing their global health impact, several members of the ESKAPE group, particularly *A. baumannii*, *P. aeruginosa*, and *K. pneumonia*, have been classified as critical priority pathogens by the WHO for the development of new antibiotics [13]. This designation reflects the urgent need for innovative therapeutic strategies, including alternative approaches such as bacteriophage therapy, antimicrobial peptides (AMPs), and vaccine development.

The substrate redundancy and broad specificity of efflux pumps pose significant challenges for inhibitor development, although targeting these systems remains a promising strategy to restore antibiotic efficacy [14,15]. As summarized in Table 1, several recently developed agents specifically address the intrinsic resistance mechanisms of ESKAPE pathogens, such as cefiderocol, which exploits a siderophore-mediated uptake pathway to overcome outer membrane impermeability in *A. baumannii*, while combinations of sulbactam–durlobactam are designed to inhibit β-lactamases and restore the activity of β-lactam antibiotics against carbapenem-resistant strains (Table 1) [16].

Decreased permeability resulting from alterations in outer membrane porins contributes to reduced antibiotic uptake, particularly in Gram-negative bacteria. Mutations, phase variation, or transcriptional downregulation of major porins such as OmpF and OmpC can limit the diffusion of hydrophilic antibiotics, including β-lactams and fluoroquinolones, thereby contributing to resistance phenotypes [14]. Recent studies suggest that synergistic interactions between efflux pump overexpression and decreased outer membrane permeability can further enhance resistance by limiting intracellular antibiotic accumulation [33]. For example, Li et al. reported that the combination of efflux activity and porin loss in *K. pneumoniae* was associated with reduced susceptibility to carbapenems and fluoroquinolones. However, these findings are largely derived from controlled experimental systems, and their quantitative contribution to resistance in clinically heterogeneous isolates remains insufficiently defined. In particular, the relative importance of this synergy appears to vary depending on the genetic background, regulatory networks, and environmental conditions, raising questions about its generalizability across pathogens. Nevertheless, porin loss alone is often insufficient to confer high-level resistance and typically acts in concert with other mechanisms, such as β-lactamase production or efflux pump activation. Porin modifications may incur fitness costs, although compensatory adaptations can partially offset these disadvantages, complicating predictions of bacterial persistence in vivo [33].

**Figure 2:**
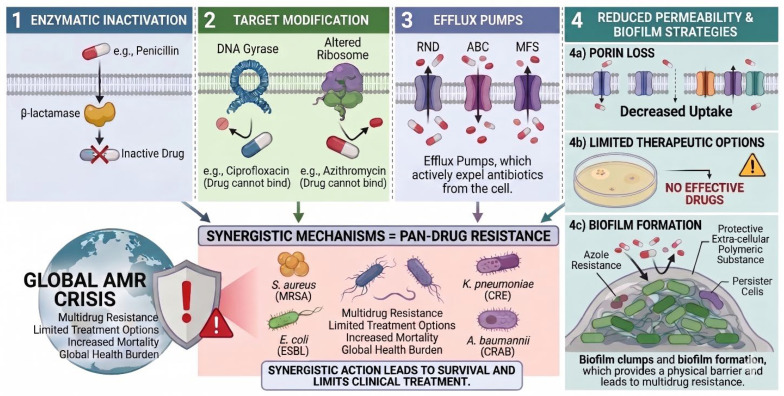
Principal mechanisms of antibiotic resistance in bacteria. The diagram illustrates the primary pathways through which bacteria develop resistance to antimicrobial agents. These include: (1) Enzymatic Inactivation, where enzymes like β-lactamase degrade the drug; (2) Target Modification, involving structural changes in DNA gyrase or ribosomes; (3) Efflux Pumps, which actively expel antibiotics from the cell; (4) Reduced Permeability, caused by the loss of porin channels; and biofilm formation, which provides a physical barrier and leads to multidrug resistance. The synergistic action of these mechanisms significantly enhances bacterial survival and limits clinical treatment options.

The broad substrate specificity and redundancy of efflux systems further complicate inhibitor development, and despite their conceptual appeal, clinically effective EPIs remain limited [34]. Importantly, resistance mechanisms rarely operate in isolation; rather, they coexist within complex and dynamic networks that differ substantially across strains and clinical contexts. While enzymatic inactivation, target modification, active efflux, and reduced permeability are frequently described as synergistic, their interactions are not uniformly additive and may be context dependent. This variability remains incompletely resolved in the current literature and continues to challenge the design of effective therapeutic strategies. Collectively, this multilayered resistance landscape significantly complicates the clinical management of infections (Figure 2).

## 3. The Evolving Antibiotic Pipeline: From Next-Generation Analogues to Novel Scaffolds

**Figure 3:**
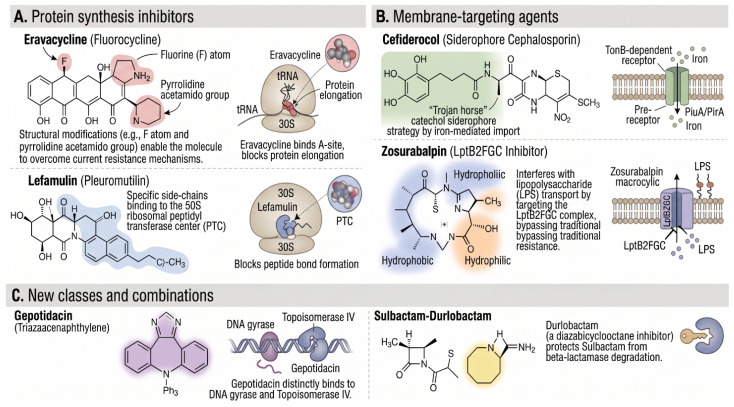
Structural classifications and mechanisms of action of next-generation antibiotics. (**A**) Protein synthesis inhibitors. Eravacycline, a fluorocycline derivative, contains characteristic structural modifications, including a fluorine atom and a pyrrolidine acetamido group (highlighted in red), which enable it to overcome established resistance mechanisms by inhibiting protein elongation at the 30S ribosomal A-site. Lefamulin, a pleuromutilin antibiotic, binds directly to the peptidyl transferase center (PTC) of the 50S ribosomal subunit through specific side-chain interactions (highlighted in blue), thereby suppressing bacterial protein synthesis. (**B**) Membrane-targeting agents. Cefiderocol, a siderophore cephalosporin, utilizes a catechol-containing siderophore moiety (green background) to facilitate “Trojan horse” iron-mediated uptake through TonB-dependent receptors, such as PiuA and PirA. Zosurabalpin, a macrocyclic antimicrobial agent, disrupts lipopolysaccharide (LPS) transport by targeting the LptB_2_FGC complex through its distinct hydrophobic and hydrophilic domains, thereby bypassing conventional resistance pathways. (**C**) Novel antibiotic classes and combination therapies. Gepotidacin, a triazaacenaphthylene antibacterial agent, represents a new antibiotic class that selectively inhibits both DNA gyrase and topoisomerase IV through a unique binding mechanism. The combination therapy sulbactam–durlobactam employs the diazabicyclooctane (DBO) core of durlobactam (highlighted in yellow) to protect the β-lactam scaffold from enzymatic degradation, thereby restoring antibacterial activity against resistant pathogens. LPS, lipopolysaccharide; PTC, peptidyl transferase center; DBO, diazabicyclooctane; tRNA, transfer RNA.

The antibiotic development pipeline has experienced a resurgence after decades of stagnation, fueled by novel funding initiatives and innovative approaches. To contextualize the urgent need for truly novel antibiotics, it is crucial to first examine recent clinical approvals, which predominantly consist of advanced analogues of existing classes. Recent clinical trials have advanced several next-generation antibiotics designed to overcome resistance mechanisms and improve safety profiles (Figure 3). However, a critical examination of the clinical and commercial trajectory of molecules developed against AMR over the past five years reveals a landscape characterized by both promising scientific breakthroughs and profound scientific, economic, and regulatory challenges. Notably, eravacycline, a fluorocycline, demonstrated non-inferiority to standard treatments in phase 3 trials for complicated intra-abdominal infections, exhibiting efficacy against multidrug-resistant (MDR) Gram-negative and Gram-positive pathogens with a favorable tolerability profile [35]. Similarly, lefamulin, a pleuromutilin antibiotic, displayed clinical response rates comparable to moxifloxacin in community-acquired pneumonia trials, including infections caused by atypical pathogens [36]. Nevertheless, the fundamental critique underlying these “successful” molecules is that they are largely next-generation analogues of existing antibiotic classes (e.g., tetracyclines, pleuromutilins). This inherent reliance on established scaffolds carries the risk of rapid evolutionary adaptation through cross-resistance mechanisms, such as target site mutations or efflux pump upregulation. Furthermore, despite securing clinical approval, the stewardship-driven practice of holding these molecules as “reserve drugs” has led to the commercial failure of their developing biotechnology companies (e.g., Achaogen, Tetraphase).

Over the last two decades, several new antibiotic classes have emerged, including cyclic lipopeptides (e.g., daptomycin), glycylcyclines (e.g., tigecycline), oxazolidinones (e.g., linezolid), and lipiarmycins (e.g., fidaxomicin), each targeting distinct bacterial processes such as cell membrane integrity or protein synthesis [37,38,39,40]. Conversely, over the past five years, the scarcity of new molecules capable of penetrating the Gram-negative outer membrane barrier has emerged as a primary point of failure. Numerous candidate molecules have been shelved during phase 2 and phase 3 trials due to nephrotoxicity, inadequate in vivo pharmacokinetics, or an inability to surpass the non-inferiority margin against standard therapies (e.g., carbapenems). In contrast, the recent clinical approvals of agents like cefiderocol (a siderophore cephalosporin) and sulbactam-durlobactam against MDR *A. baumannii* and *P. aeruginosa* demonstrate that specific, targeted successes in treating highly resistant infections remain achievable.

Currently, promising broad-spectrum candidates such as nafithromycin and gepotidacin are progressing through late-stage trials, targeting respiratory and urinary tract infections, respectively [41]. Gepotidacin represents one of the most critical and rare success stories of the recent era, possessing the potential to be the first molecule with an entirely novel mechanism of action (a triazaacenaphthylene-class topoisomerase inhibitor) approved for Gram-negative urologic infections in decades.

Looking ahead, entirely novel chemical entities such as zosurabalpin, which aim to address urgent threats like MDR *A. baumannii*, hold the potential to fill critical therapeutic gaps by 2030 and will undoubtedly shape future clinical guidelines [1,2,3]. The example of zosurabalpin, which uniquely targets the LptB2FGC complex, underscores the strategic imperative in efforts to overcome AMR: the field must focus not merely on improved analogues of existing antibiotic classes, but decisively on entirely novel chemical scaffolds that attack the unique physiology of Gram-negative pathogens and are devoid of pre-existing selective resistance pressure.

## 4. Lariocidin: Mechanism of Action, Resistance Potential, and Future Clinical Perspectives

While the recently approved antimicrobials and combination therapies detailed in the preceding section provide critical immediate lines of defense against ESKAPE pathogens, a fundamental limitation remains that most of these agents are structural analogues of established antibiotic classes, carrying an inherent risk of rapid cross-resistance adaptation [42]. To build a sustainable, long-term therapeutic pipeline against AMR, the discovery paradigm must transition away from modifying existing scaffolds and move decisively toward entirely novel chemical entities with unprecedented modes of action [43]. In this framework, lariocidin (LAR) serves as a pivotal representative case study. Although it is currently a preclinical candidate, this ribosomally synthesized and post-translationally modified peptide (RiPP) features a unique, tightly knotted lasso topology and targets a previously unexploited binding pocket on the 30S small ribosomal subunit [44,45]. Incorporating a dedicated analysis of lariocidin in this review is therefore fundamentally justified; it provides a mechanistic blueprint for future drug design, illustrating how novel structural platforms can completely circumvent pre-existing target modifications and efflux networks to outpace bacterial evolution.

**Figure 4:**
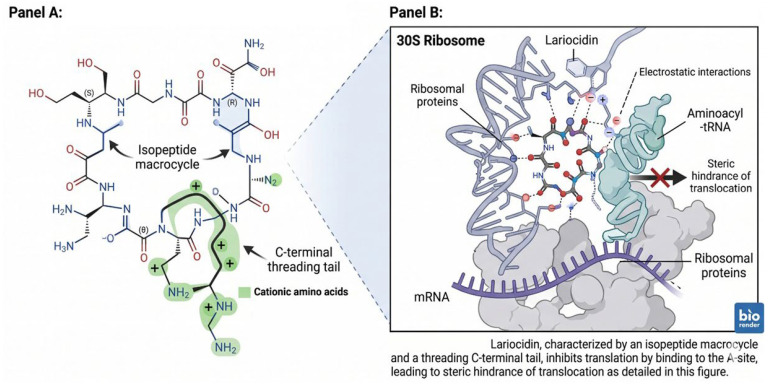
Mechanistic schematic illustration of lariocidin structure and ribosomal inhibition mechanism. (**A**) Chemical Structure and “Lasso” Topology; (**B**) Molecular Interaction with the Ribosome A-site.

Isolated from *Paenibacillus* species found in soil, this novel discovery exhibits potent activity against both Gram-positive and Gram-negative bacteria, including multidrug-resistant *E. coli* strains [46]. Crucially, preclinical studies indicate minimal cytotoxicity toward human cells, underscoring its therapeutic potential. Because LAR’s ribosomal targeting diverges from classical antibiotics such as aminoglycosides and macrolides, it represents a paradigm shift in structurally constrained antimicrobial chemotypes. This architectural stability is achieved via a covalent isopeptide bond linking the N-terminal amine of Serine-1 to the carboxyl side chain of Aspartate-8, creating a rigid macrocycle through which its highly basic C-terminal tail is sterically threaded. Structural data indicates that LAR forms highly specific hydrogen bonds and electrostatic interactions with both the 16S ribosomal RNA and the incoming aminoacyl-tRNA at the A-site. This dual interaction arrests mRNA translation by sterically hindering ribosomal translocation while simultaneously inducing critical miscoding events (Figure 4) [47].

**Figure 5:**
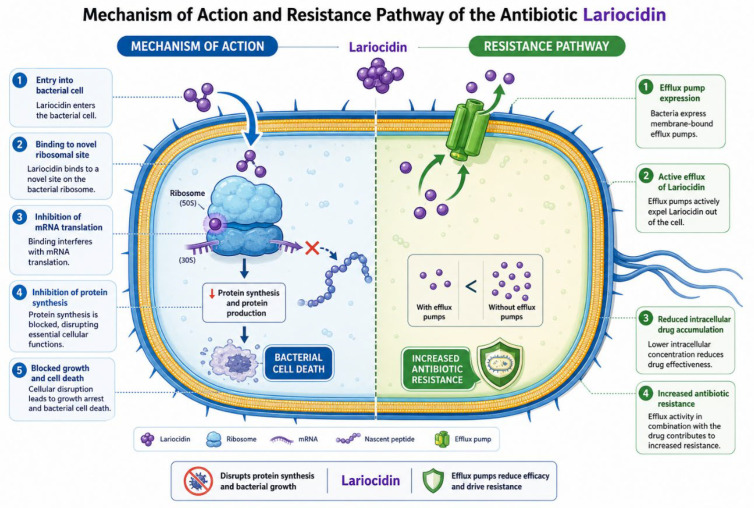
Mechanism of action and resistance pathway of antibiotic lariocidin. Lariocidin enters the bacterial cell and binds to a previously uncharacterized site on the ribosome, thereby disrupting mRNA translation and inhibiting protein synthesis, ultimately leading to growth arrest and cell death. In parallel, membrane-associated efflux pumps can actively expel the antibiotic, reduce its intracellular accumulation and contribute to antimicrobial resistance.

The broad-spectrum efficacy of lariocidin, encompassing pathogens prioritized by the WHO for urgent antimicrobial development, positions it as a critical candidate in the antibiotic pipeline. The clinical translation of next-generation antibiotics like lariocidin requires rigorous evaluation through phased clinical trials to establish safety, pharmacokinetics, and efficacy in human populations. While preclinical data are promising, comprehensive clinical trials are not yet complete [37,48]. Furthermore, integrating novel agents into antibiotic use management programs is crucial to mitigate rapid resistance development [47,48,49]. Despite its unique ribosomal targeting, the potential for resistance development cannot be excluded; under selective pressure, bacteria may evolve de novo mechanisms—such as specific efflux pump upregulations—capable of reducing intracellular drug accumulation (Figure 5).

Furthermore, potential challenges related to in vivo pharmacokinetics, including tissue penetration and stability, as well as unintended impacts on human microbiota, should be carefully considered when evaluating its clinical applicability. Lariocidin represents a breakthrough by targeting novel ribosomal sites; the next decade will likely shift toward “smart” antibiotics that are only activated by specific bacterial enzymes. This would theoretically eliminate “collateral damage” to the human microbiome, which is a major drawback of current broad-spectrum therapies.

## 5. Next-Generation Biotherapeutics: CRISPR-Cas and Beyond

**Figure 6:**
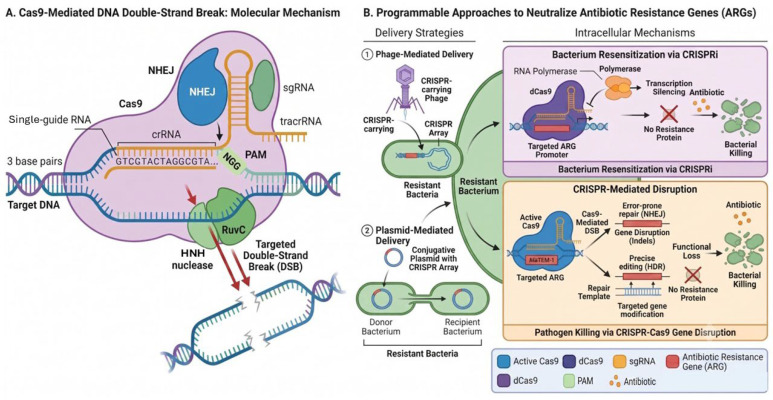
CRISPR/Cas-based strategies to neutralize antibiotic resistance. (**A**) Cas9-Mediated DNA Double-Strand Break: Molecular Mechanism is a detailed close-up of the Cas9 ribonucleoprotein (RNP) complex, including a focused inset that highlights the chemical nature of the phosphodiester bond cleavage with labels for chemical groups. (**B**) Programmable Approaches to Neutralize Antibiotic Resistance Genes (ARGs) illustrates the overall strategies for delivering these tools and the two main intracellular mechanisms for disabling resistance determinants: CRISPR interference (CRISPRi) using catalytically inactive dCas9, and gene disruption using active Cas9. This panel shows the flow from horizontal delivery (via phages and plasmids) to bacterial resensitization and subsequent killing when exposed to antibiotics.

Recent advances in molecular biology have positioned CRISPR/Cas-based technologies as promising tools to combat AMR through sequence-specific targeting of resistance determinants. Mechanistically, CRISPR-Cas9 enables precise modification of DNA sequences by introducing site-specific double-strand breaks, guided by a synthetic single-guide RNA (sgRNA) and requiring a Protospacer Adjacent Motif (PAM) [50,51,52]. Alternatively, CRISPR interference (CRISPRi) employs catalytically inactive Cas proteins (e.g., dCas9) to repress the transcription of antibiotic resistance genes (ARGs) without inducing DNA cleavage, thereby potentially reducing the selective pressure for escape mutations (Figure 6) [53]. Engineered CRISPR-carrying phages and CRISPR-Cas13a antimicrobial platforms have also demonstrated effectiveness against MDR bacteria, offering high specificity while preserving the beneficial microbiota [54,55]. By targeting specific ARGs, CRISPR systems have successfully resensitized bacteria to conventional antibiotics in various experimental models. For instance, the targeted removal or disruption of the *mecA* gene—which encodes the low-affinity alternative penicillin-binding protein PBP2a—in methicillin-resistant *S. aureus* (MRSA) eliminates the bacterium’s primary resistance mechanism, restoring susceptibility to β-lactam antibiotics [56]. Similarly, CRISPR-Cas9 systems have been used to silence the *AmpC* β-lactamase gene in *A. baumannii* via natural transformation (Figure 6) [53]. Beyond direct cleavage, experimental studies demonstrate that CRISPRi-mediated silencing of ARGs in *E. coli* can increase susceptibility to multiple antibiotic classes, including tetracyclines, β-lactams, and polymyxins [57].

Despite these conceptual and experimental advances, the clinical translation of CRISPR-Cas systems is constrained by severe in vivo limitations [54,57]. A primary bottleneck is delivery; the reliance on bacteriophage vectors or conjugative plasmids introduces challenges regarding delivery efficiency, host range limitations, and the risk of horizontal gene transfer with unpredictable consequences for microbial communities [58]. Furthermore, immunogenicity poses a critical barrier. A substantial portion of the human population possesses pre-existing adaptive immunity against common Cas endonucleases (e.g., from *S. pyogenes* or *S. aureus*), and host immune responses against bacteriophage vectors can trigger systemic inflammation and rapid clearance of the therapeutic agent [59]. Additionally, the immense selective pressure exerted by DNA cleavage inherently risks the rapid selection of escape mutants; a single nucleotide polymorphism within the PAM site or the sgRNA seed region can render the bacteria completely refractory to the intervention. Finally, the deployment of genetically engineered phages classifies these interventions as genetically modified organisms (GMOs), introducing profound regulatory, ecological, and safety hurdles [60]. Consequently, CRISPR-based approaches are currently more likely to function as adjunctive tools that complement existing therapies rather than standalone solutions [61,62]. To address these limitations and broaden the therapeutic arsenal, the scope of next generation biotherapeutics actively includes several other highly targeted strategies [50,51]. Phage endolysins—bacteriophage-derived enzymes that rapidly hydrolyze the bacterial peptidoglycan cell wall—exhibit potent, “lysis-from-without” bactericidal activity, particularly against Gram-positive pathogens, with a markedly low propensity for resistance development. Concurrently, engineered bacteriophages are being optimized to overcome natural host-range limitations and actively degrade extracellular biofilm matrices [63]. Bacteriocins, highly specific ribosomally synthesized antimicrobial peptides produced by bacteria, are also being harnessed to selectively target resistant strains without disrupting the commensal microbiome. Finally, the application of antibacterial monoclonal antibodies (mAbs) offers a sophisticated precision medicine approach. By targeting specific bacterial virulence factors, toxins, or surface antigens, mAbs facilitate immune-mediated pathogen clearance and neutralize pathogenesis, serving as a critical non-antibiotic adjunct in the control of multidrug-resistant infections [64].

## 6. Precision Engineering of Nanocarriers for Targeted Therapy in AMR

**Figure 7:**
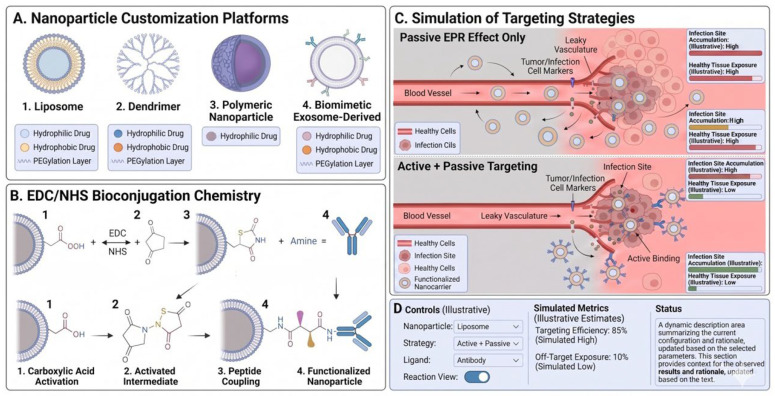
Precision molecular engineering of nanocarriers for targeted therapy. (**A**) Schematic representation of customizable nanoparticle platforms, including liposomes, dendrimers, polymeric nanoparticles, and biomimetic exosome-derived vesicles for therapeutic delivery. (**B**) Illustration of EDC/NHS-mediated bioconjugation chemistry used for covalent attachment of targeting ligands onto nanoparticle surfaces. (**C**) Comparison of passive EPR-based targeting and ligand-mediated active targeting strategies for enhancing nanoparticle accumulation at infection sites. (**D**) Simulated control parameters and predicted targeting metrics demonstrating the influence of nanoparticle design on delivery efficiency and off-target exposure.

Nanoparticle drug delivery systems (DDS) have revolutionized traditional therapeutic administration by improving the solubility, bioavailability, and controlled release of antimicrobial agents [65,66]. Various platforms—including liposomes, dendrimers, polymeric formulations, inorganic nanoparticles, and biomimetic exosome-derived carriers—can be engineered to overcome biological barriers and address patient heterogeneity. These systems maximize drug concentration at infection sites while minimizing systemic off-target toxicity by exploiting both passive targeting, via the enhanced permeability and retention (EPR) effect, and active targeting. From a molecular engineering perspective, active targeting is often achieved using robust bioconjugation techniques, such as EDC/NHS (1-ethyl-3-(3-dimethylaminopropyl) carbodiimide/N-hydroxysuccinimide) coupling chemistry, to covalently immobilize specific targeting ligands, aptamers, or antimicrobial peptides onto the nanocarrier surface (Figure 7) [65].

The impact of biofilm formation on antimicrobial resistance, particularly among ESKAPE pathogens, represents one of the most formidable challenges in modern infectious disease management. Biofilms are complex, surface-attached microbial communities encased in a self-produced extracellular polymeric substance (EPS) matrix [67]. This robust physical and chemical barrier actively sequesters and neutralizes conventional antibiotics before they can reach their intracellular targets. Furthermore, the hypoxic and nutrient-deprived microenvironment deep within the biofilm architecture promotes the transition of a bacterial subpopulation into metabolically dormant ‘persister cells’ [68]. Because traditional bactericidal antibiotics rely on active cellular division to disrupt cell wall synthesis or DNA replication, these persister cells remain highly tolerant to treatment, surviving the initial antibiotic onslaught to seed recurrent, recalcitrant infections [69].

**Figure 8:**
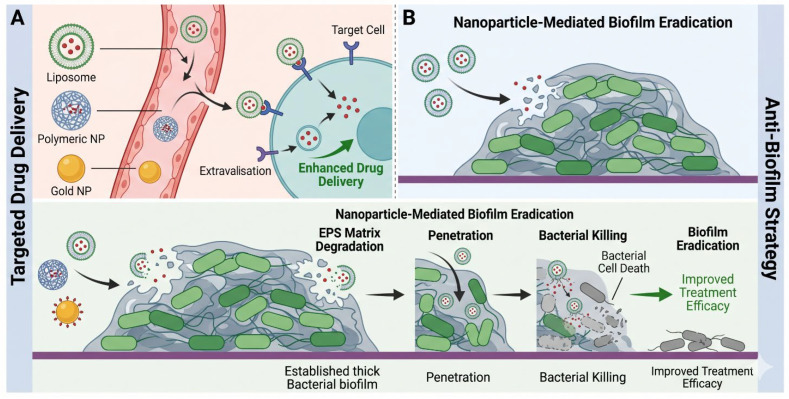
Nanotechnology-based strategies for drug delivery and biofilm control. This schematic highlights the applications of nanotechnology in drug delivery and combating bacterial biofilms for improved therapeutic outcomes. (**A**) Under targeted drug delivery, various nanoparticles (liposomes, polymeric nanoparticles, gold nanoparticles) are shown for delivering drugs precisely to target cells, leading to enhanced drug delivery. (**B**) The anti-biofilm section illustrates how nanoparticles can disrupt the biofilm matrix, degrade it, penetrate deep into the biofilm structure, and cause bacterial cell death, effectively eradicating the biofilm and improving overall treatment efficacy.

To overcome these intrinsic biofilm defenses, precision-engineered nanocarriers are increasingly utilized to disrupt the EPS matrix and achieve potent synergy with conventional antibiotics. Nanoparticles can be surface-functionalized with matrix-degrading enzymes or designed with specific cationic charges to deeply penetrate the negatively charged EPS layer [70]. Once embedded, these nanocarriers facilitate a highly localized, sustained release of antibiotics, exposing both metabolically active bacteria and dormant persister cells to overwhelming drug concentrations. Moreover, co-delivering antibiotics with inorganic nanoparticles (such as silver or gold) produces a multi-target synergistic effect; the nanoparticles induce localized reactive oxygen species (ROS) generation and physical membrane disruption, which dramatically lowers the minimum inhibitory concentration (MIC) of the paired antibiotic. By overcoming diffusion barriers and protecting drugs from premature degradation, this nanoparticle-antibiotic synergy effectively resensitizes multidrug-resistant populations to previously compromised antimicrobial agents, presenting a highly viable new-generation antibiofilm therapy (Figure 8) [70,71].

Despite these highly promising in vitro and experimental results, the clinical translation of nanomedicines remains severely limited by the challenges associated with the so-called “Valley of Death” [72]. A major barrier is the rapid in vivo recognition and clearance of these particles by the mononuclear phagocyte system, which significantly reduces their circulation time and therapeutic efficacy at the infected tissues [65]. Furthermore, concerns regarding systemic toxicity persist, particularly regarding metal-based nanozymes that may accumulate in vital organs such as the liver and kidneys. Biocompatibility and structural stability also present hurdles; surface-targeting ligands may undergo degradation under physiological conditions, reducing targeting precision [73]. For biomimetic platforms, such as exosome-derived delivery systems, large-scale production is hindered by low isolation yields, batch-to-batch variability, and difficulties in achieving standardized purification and long-term stability. Addressing these pharmacokinetic and manufacturing limitations through improved stealth strategies and functionalization is critical to ensuring the safe and reliable integration of targeted nanotherapies into clinical practice [74].

## 7. Ongoing Antibacterial Approaches

Bacteriophage therapy has re-emerged as a promising alternative to conventional antibiotics amid escalating antimicrobial resistance. Recent studies have demonstrated the efficacy of engineered and naturally isolated phages in selectively targeting multidrug-resistant bacterial strains, including *P. aeruginosa* and *S. aureus*. Advances in phage formulation and delivery have improved stability and host specificity, while combination therapies with antibiotics show synergistic effects that reduce bacterial load and biofilm formation. Clinical case reports and ongoing trials underscore phage therapy’s potential to complement or replace antibiotics, particularly in recalcitrant infections where traditional treatments fail [75].

AMPs continue to attract significant attention as broad-spectrum agents capable of disrupting bacterial membranes and modulating host immune responses. Recent research highlights novel synthetic and naturally derived AMPs with enhanced stability, reduced cytotoxicity, and improved efficacy against resistant pathogens. Mechanistic insights reveal that AMPs not only permeabilize microbial membranes but also interfere with intracellular targets and biofilm integrity. Furthermore, AMPs can act as immunomodulators, promoting beneficial inflammatory responses and tissue repair, positioning them as dual-function therapeutics in infectious disease management. Immune modulation strategies have gained traction as adjunct or standalone therapies to augment host resistance against infections [76].

Recent advances involve the use of cytokine therapies, immune checkpoint inhibitors, and nanotechnology-enabled delivery systems to fine-tune immune responses. These approaches aim to enhance pathogen clearance while minimizing tissue damage caused by excessive inflammation. Nanotechnology is moving beyond simple drug delivery toward diagnostic and therapeutic nanoparticles that simultaneously diagnose infection and deliver targeted antimicrobial interventions. The integration of artificial intelligence (AI) with nanoparticle surface engineering will likely lead to “bio-homing” carriers that can navigate the dense extracellular matrix of chronic biofilms, a feat currently impossible for free-form drugs [72]. Collectively, these ongoing approaches—alongside the novel antibiotics, diagnostic tools, and genetic interventions discussed throughout this review—form a multi-layered defense strategy. A comprehensive summary of these next-generation technologies, including their developmental stages, advantages, and limitations, is provided in Table 2.

## 8. Future Perspectives, Diagnostics and Global Surveillance

Effective management of antimicrobial resistance increasingly relies on rapid, precise diagnostic tools and comprehensive surveillance systems. At the molecular level, the development of electronic aptasensors and targeted lateral flow assays offers diagnostic agility. By leveraging the specific three-dimensional folding architectures of oligonucleotide aptamers, these biosensors can achieve highly sensitive, real-time detection of bacterial antigens and resistance biomarkers. This molecular surveillance facilitates early identification of emerging resistance patterns, guiding clinical decision-making and public health interventions. Furthermore, stewardship programs that combine diagnostic stewardship with optimized antibiotic prescribing have shown promise in slowing resistance evolution. The dynamic interplay between pathogen adaptation and therapeutic pressure necessitates continuous monitoring frameworks supported by global data sharing and harmonized protocols [88,89].

Addressing antimicrobial resistance demands coordinated global health policies that balance access, innovation, and stewardship. Recent policy frameworks advocate for sustainable antibiotic development incentives, equitable access to novel therapies, and stringent regulation of antibiotic use in human and veterinary medicine. The WHO Global Action Plan highlights the importance of integrating surveillance, infection prevention, and awareness campaigns to reduce inappropriate antibiotic consumption. Moreover, international collaborations such as CARB-X and the Global Antibiotic Research and Development Partnership are accelerating research pipelines and facilitating access in low- and middle-income countries. These efforts aim to mitigate the global burden of resistant infections while fostering responsible antibiotic deployment to preserve efficacy for future generations [88,89].

AI, particularly deep learning approaches, is increasingly transforming antibiotic discovery by enabling the rapid identification and design of novel chemical scaffolds with antimicrobial activity. Advanced algorithms can screen vast chemical spaces far beyond the reach of traditional methods, predict structure–activity relationships, and optimize lead compounds with improved efficacy and reduced toxicity. Notably, AI-driven platforms have already demonstrated success in identifying entirely new classes of antibiotics by learning from large-scale biochemical and genomic datasets, offering a promising strategy to overcome the stagnation in conventional drug discovery pipelines [90]. Traditional “trial and error” method of drug discovery is undergoing a change in basic assumptions. We are entering an era where deep learning models can predict the “resistance trajectory” of a molecule before it even enters Phase I trials, allowing researchers to design “resistance-resistant” scaffolds from the outset. In addition, AI can be integrated with omics data and systems biology approaches to better understand host–pathogen interactions and predict resistance evolution. Such models may identify mutational pathways likely to emerge under antimicrobial pressure, allowing researchers to anticipate resistance development, prioritize compounds with lower evolutionary vulnerability, and guide the development of precision antimicrobials [91].

For instance, the integration of AI-guided molecular design with advanced aptamer technology holds tremendous clinical potential for managing critical ESKAPE pathogens. Recent conceptual frameworks highlight how machine learning can optimize aptamer sequences to rapidly detect specific resistance determinants, such as carbapenemases in *K. pneumoniae* or the *mecA* gene products in MRSA, directly from clinical samples via lateral flow assays [92]. Ultimately, this seamless integration of predictive algorithmic modeling, high-precision biomolecular engineering, and robust global surveillance exemplifies the multidimensional strategy required to systematically outpaced by bacterial evolution.

## 9. Conclusions

The response to AMR is entering a new phase characterized by enhanced antibiotic design and innovative alternatives. While new-generation antibiotics offer hope, their longevity depends on prudent use and complementary strategies to outpace bacterial resistance. The escalating global burden of antimicrobial resistance demands a multifaceted and sustained response. This review has highlighted the complex and interconnected resistance mechanisms—enzymatic inactivation, target modification, efflux, and biofilm formation—that challenge antibiotic therapy. New-generation agents such as lariocidin represent critical additions to the therapeutic arsenal, offering novel mechanisms of action against resistant pathogens. Complementary strategies including CRISPR-based gene silencing, nanotechnology-enabled drug delivery, phage therapy, and AMPs further expand the options available to clinicians and researchers. The clinical translation of these innovations requires rigorous evaluation through well-designed trials, robust global surveillance frameworks, and coordinated stewardship programs. Sustained investment in research, equitable access to novel therapies, and responsible antimicrobial use are the cornerstones of any durable solution to the AMR crisis.

## Figures and Tables

**Table 1:** Recent antimicrobial strategies against ESKAPE pathogens.

Pathogen	Antimicrobial Compound/Strategy	Mechanism of Action	Key Findings	References
*E. faecium* (VRE)	Daptomycin + β-lactam combination	Daptomycin and β-lactams act synergistically to restore susceptibility and limit resistance in VRE.	The combination shows synergistic bactericidal activity against VRE, lowers daptomycin MIC up to fourfold, outperforms monotherapy in endocarditis models, and is supported by 2021 IDSA guidance.	[17,18,19]
*S. aureus* (MRSA)	Ceftaroline fosamil (5th-gen cephalosporin)	A prodrug converted to ceftaroline binds PBP2a with high affinity, inhibiting cell wall synthesis and overcoming MRSA resistance.	Ceftaroline achieved clinical cure in MRSA skin infections, was non-inferior to vancomycin, and showed strong global activity including against resistant strains.	[20]
*S. aureus* (MRSA)	Bacteriophage therapy + vancomycin (compassionate use)	Phages lyse bacteria and disrupt biofilms, enhancing antibiotic penetration, while phage–vancomycin synergy targets cell wall and membrane to reduce resistance.	The Sb-1–vancomycin combination markedly reduces MRSA biofilms and has cleared refractory bacteremia, with clinical evidence reviewed.	[21]
*K. pneumoniae* (KPC+, CR-Kp)	Ceftazidime-avibactam (CAZ-AVI)	Ceftazidime targets PBPs while avibactam inhibits class A, C, and some D β-lactamases, but not metallo-β-lactamases.	Ceftazidime-avibactam is superior to colistin/polymyxin regimens for CR-Kp bacteremia with ~71% efficacy, though KPC mutations can drive 5–15% resistance in endemic regions.	[22,23]
*K. pneumoniae* (NDM+, MBL-producing)	Aztreonam-avibactam (ATM-AVI; Emblaveo™)	Aztreonam targets PBP3 and resists MBLs, while avibactam blocks co-produced serine β-lactamases, restoring activity against NDM/VIM + KPC producers.	Aztreonam-avibactam showed non-inferior cure to meropenem in Phase 3 trials with lower HAP-VAP mortality and FDA-approved in 2025 for infections.	[24]
*K. pneumoniae* (Imipenem-non-susceptible)	Imipenem-cilastatin-relebactam (Recarbrio™)	Imipenem inhibits PBPs, relebactam blocks class A/C β-lactamases, and cilastatin prevents renal degradation, but it remains inactive against metallo-β-lactamases.	Imipenem-cilastatin-relebactam showed comparable or improved clinical outcomes versus comparators with better renal safety and activity against KPC-producing isolates in RESTORE trials.	[25,26]
*A. baumannii* (CRAB)	Sulbactam-durlobactam (SUL-DUR; Xacduro™)	Sulbactam directly targets PBPs in *A. baumannii*, while durlobactam protects it from key β-lactamases, restoring activity against CRAB without needing a carbapenem.	Sulbactam-durlobactam reduced mortality vs. colistin in CRAB pneumonia with markedly lower nephrotoxicity and was FDA-approved in 2023 for HAP/VAP.	[26]
*A. baumannii* (XDR/CRAB)	Cefiderocol (siderophore cephalosporin)	A siderophore–cephalosporin conjugate uses iron-uptake transporters for active entry, resists β-lactamases, inhibits PBP3, and overcomes Gram-negative permeability and efflux barriers.	Cefiderocol shows high activity against XDR Gram-negatives and improved mortality vs. imipenem in pneumonia, though similar cure rates in CRAB infections.	[20,27]
*P. aeruginosa* (MDR)	Ceftolozane-tazobactam (C/T; Zerbaxa™)	Ceftolozane/tazobactam has strong PBP3 activity and β-lactamase inhibition, improving efficacy against resistant *P. aeruginosa* by overcoming permeability, *AmpC*, and efflux mechanisms.	Ceftolozane-tazobactam was non-inferior to meropenem for HAP/VAP and shows strong clinical success against MDR/XDR *P. aeruginosa*.	[28,29]
*P. aeruginosa* (MDR/XDR)	Cefiderocol	A siderophore cephalosporin uses iron transport to enter bacteria, evades permeability and efflux barriers, resists β-lactamases, and inhibits PBP3 with high intracellular activity.	Cefiderocol shows near-complete activity against MDR/XDR *P. aeruginosa* and improved 28-day survival vs. meropenem in nosocomial pneumonia, including efflux-overexpressing strains.	[30,31]
*Enterobacter cloacae* complex (*AmpC*, *CR*)	Meropenem-vaborbactam (Vabomere™)	Meropenem-vaborbactam combines PBP inhibition with selective KPC/ESBL β-lactamase inhibition but remains inactive against metallo-β-lactamases and OXA carbapenemases.	Meropenem-vaborbactam showed higher clinical cure and lower mortality than best available therapy in CR-Enterobacterales infections, with confirmed real-world effectiveness against KPC producers.	[27,32]

Abbreviations: CRAB: carbapenem-resistant *A. baumannii*; CR-Kp: carbapenem-resistant *K. pneumoniae*; HAP: hospital-acquired pneumonia; MDR: multidrug-resistant; MIC: minimum inhibitory concentration; MRSA: methicillin-resistant *S. aureus*; NDM: New Delhi metallo-β-lactamase; PBP: penicillin-binding protein; VAP: ventilator-associated pneumonia; VRE: vancomycin-resistant Enterococcus; XDR: extensively drug-resistant; ACM: all-cause mortality; BSI: bloodstream infection; KPC: *K. pneumoniae* carbapenemase; ESBL: Extended-spectrum β-lactamase; MBL: Metallo-β-lactamase.

**Table 2:** Next-Generation Technologies Against Antimicrobial Resistance.

Technology	Development Stage/Phase	Main Advantage	Main Limitation	References
Next-generation antibiotics	Preclinical to late clinical, depending on class	Can directly kill resistant bacteria and fit existing prescribing workflows	Discovery is slow, resistance can still emerge, and many candidates fail before approval	[77]
Rapid molecular AST/point-of-care susceptibility testing	Prototype to clinical implementation	Speeds up correct treatment and reduces unnecessary broad-spectrum antibiotic use	Needs validation across pathogens, settings, and sample types; performance can vary	[78]
Bacteriophage and Endolysins	Experimental to early clinical use	High strain specificity; works synergistically with existing antibiotics; endolysins offer rapid “lysis-from-without” with low resistance rates.	Narrow host range; potential to trigger systemic inflammation; complex regulatory frameworks and limited standardization.	[75,79]
CRISPR-based antimicrobials	Mostly preclinical, advanced proof-of-concept	Highly precise sequence-specific targeting (e.g., *mecA* gene disruption); spares host microbiota; CRISPRi minimizes selective escape pressure.	Severe in vivo delivery bottlenecks using phage vectors/plasmids; host immunogenicity to Cas proteins; strict GMO regulatory hurdles.	[53,80,81]
Anti-biofilm therapies	Preclinical to early translational	Targets biofilms, which are a major cause of tolerance and chronic infection	Biofilms are biologically diverse and hard to penetrate; often needs combination therapy	[82]
Monoclonal antibodies/immunotherapies	Clinical for selected infections, exploration for broader AMR use	Can neutralize toxins or help immune clearance without directly selecting for classical antibiotic resistance	Usually pathogen- or toxin-specific and often expensive	[83]
Microbiome-based therapies	Early clinical to exploratory	May restore colonization resistance and reduce recurrence, especially after infection or antibiotic exposure	Mechanisms are complex, and long-term safety/consistency are still being defined	[84]
Pro-Active Genetics and related gene-drive-like systems	Experimental, early research	Can spread resistance-disabling elements through bacterial populations in principle	Highly experimental, with major containment, delivery, and ethics questions	[85]
AI-guided discovery	Research and preclinical platform	Acceleration in screening vast chemical spaces; designs novel scaffolds and models resistance trajectories before synthesis.	Purely predictive and theoretical; still entirely dependent on traditional, lengthy downstream in vivo validation and clinical success.	[86]
Combination and adjuvant strategies	Clinical to translational	Can restore activity of older antibiotics by blocking resistance mechanisms or permeability barriers	Effects may be context-specific and may not generalize across organisms	[87]
AMPs	Experimental/Translational	Broad-spectrum membrane disruption; dual-function immune modulation and tissue repair.	Stability issues, potential cytotoxicity, and high production costs.	[76]
Precision Nanocarriers	Preclinical/Early translation	Overcomes biological barriers to maximize localized drug concentrations; degrades biofilm EPS matrix; reduces systemic toxicity.	Rapid in vivo clearance by the mononuclear phagocyte system; long-term organ accumulation toxicity; low isolation yields/scaling issues.	[65,71,72]
LAR	Preclinical candidate	Unique lasso topology; targets an unexploited 30S ribosomal pocket to bypass existing target modifications and efflux networks.	Lacks complete clinical trial data; selective pressure risks de novo resistance via novel efflux upregulations; in vivo stability/tissue penetration needs mapping.	[46,47]

## Data Availability

No datasets were generated or analyzed during the current study.
